# Should Implant Breakage Be Always Considered as Implant “Failure” in Spine Surgery: Analysis of Two Cases and Literature Review

**DOI:** 10.7759/cureus.15233

**Published:** 2021-05-25

**Authors:** Anuj Gupta, Kalidutta Das, Kuldeep Bansal, Harvinder Singh Chhabra, Mohit Arora

**Affiliations:** 1 Orthopedics and Spine, Triveni Ortho and Spine Center, New Delhi, IND; 2 Spine Surgery, Indian Spinal Injuries Center, New Delhi, IND; 3 Orthopedics, Indian Spinal Injuries Center, New Delhi, IND; 4 Spine Services, Indian Spinal Injuries Center, New Delhi, IND; 5 Orthopedics, Narayana Superspeciality Hospital, New Delhi, IND

**Keywords:** implant failure, implant breakage, pseudoarthrosis, spine, instrumentation

## Abstract

The advancement in the material of spinal implant and technique of spinal instrumentation has led to an increase in spine surgeries. The final desired outcome of spine surgery involving instrumentation is fusion. There is a race among implants to fail and bone to fuse. If there is a formation of pseudoarthrosis or failure to fuse then implants are bound to fail. The most common presentation of pseudoarthrosis is implant breakage. Hence, should we label every implant that has presented with breakage as a “failure”? In this article, we have discussed our experience of two cases presented to us with implant breakage but which were managed successfully with conservative methods. Both of our cases did well without any surgical intervention. We have follow-ups of seven years in one case and five years in the other. Every patient with pseudoarthrosis does not require surgical management and hence, every implant breakage should not be labeled as implant “failure”.

## Introduction

With the increasing knowledge and understanding of various disorders involving spinal columns, spine surgery has also evolved to a great extent. Also, the acceptance of spine surgery among common people has increased. All these factors have greatly increased the number of spine surgeries compared to the past.

One of the most important parts of spine surgery is instrumentation and fusion. The methods of mechanical fixation have improved over the years owing to research and development in the field of spine disorders, but the final result of most of the spine surgeries, which every spinal surgeon desires, is fusion. In the 1930s, the material used for spinal fixation was suboptimal and the incidence of implant failure was very high [1]. After the 1940s, the material and methods of instrumentation have undergone substantial changes that can withstand the repeated stress of weight-bearing, flexion, and extension until arthrodesis occurs.

The instrumentation used in spine surgery is not to replace the bony elements but to hold them during the fusion process [2]. There is a race among implants to fail and bone to unite. Implant failure can be said to occur if it ceases to perform the function for which it is inserted. Implant failure can present in many ways like migration, dislodgement, or implant fracture. The various causes of instrumentation failure can be patient-related factors, biomechanical factors, or procedure-related factors. The most common reason for implant failure is pseudoarthrosis or failure to achieve fusion [3]. The most common presentation in cases of pseudoarthrosis is rod breakage [4]. Various authors define implant failure as the breakage of a screw or rod and use both the terms interchangeably without considering clinical symptoms and signs into the picture. The gold standard to diagnose pseudoarthrosis is an operative exploration of the fusion mass, which is not achievable in every case of implant breakage, especially in developing countries, where cost is one of the biggest factors. Also, no single investigation has accuracy high enough to diagnose pseudoarthrosis. So, the biggest dilemma is: Should every rod breakage or implant breakage be considered an implant “failure” in spine surgery needing revision surgery?

## Case presentation

Case 1

**Figure 1:**
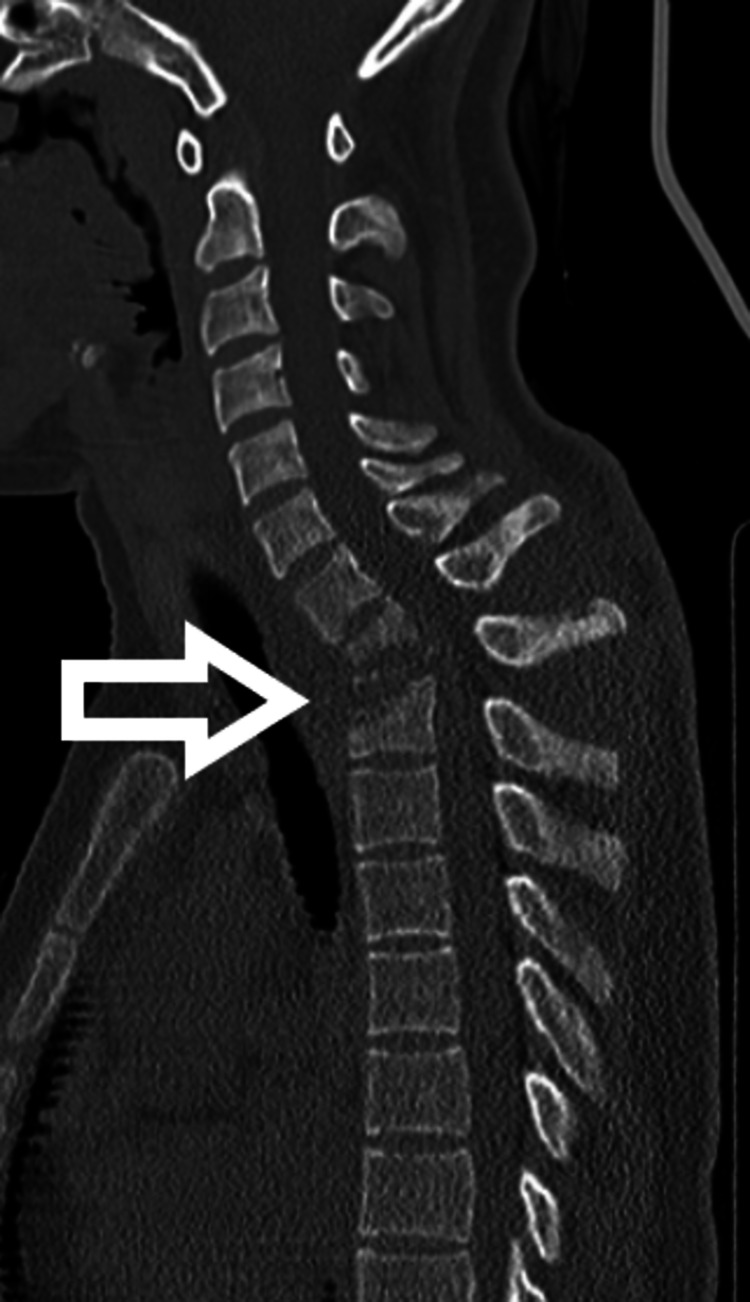
Pre-operative CT Scan showing the involved region

**Figure 2:**
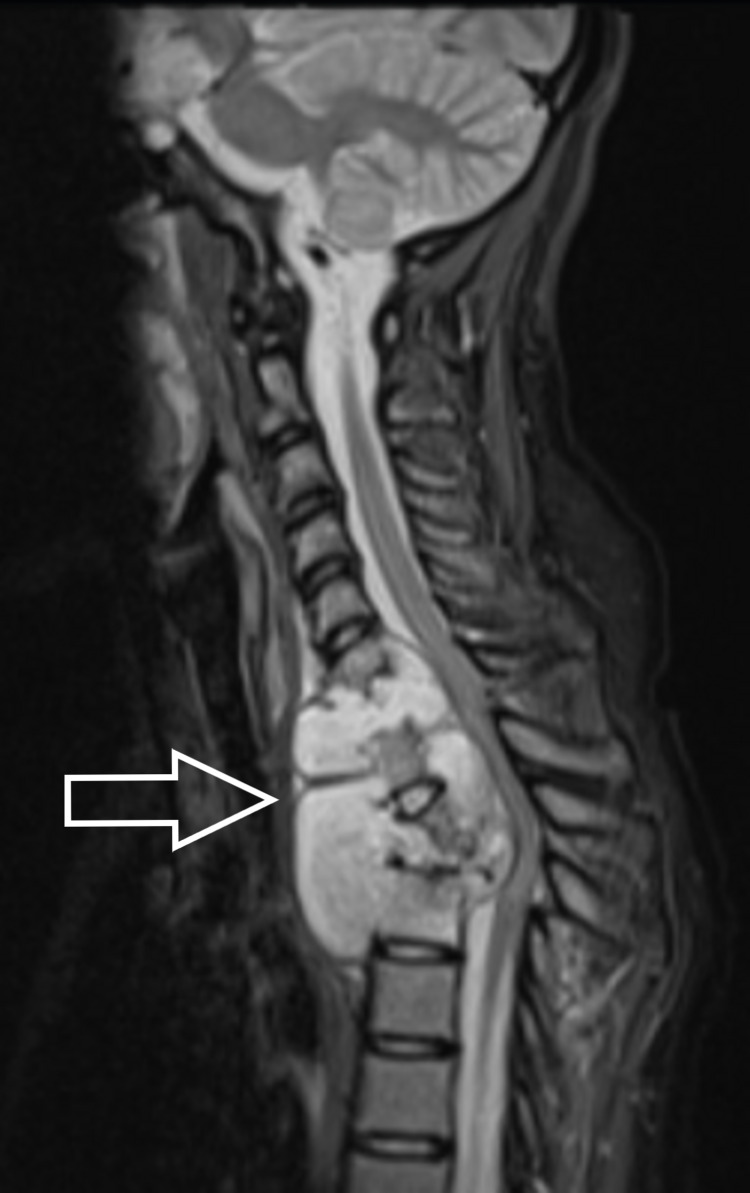
Pre-operative MRI of the involved region

A 29-year-old female presented to us with complains of neck pain and weakness in both upper limbs for six months. She also had constitutional symptoms like fever, loss of appetite, loss of weight, and night pain. On examination, she had all upper motor neuron features along with grade 4 motor power in most of the muscle groups. Her neurological level was T1. On MRI (Figures 1, 2), she had an epidural and paravertebral collection at cervico-dorsal junction with paradiscal involvement of T1-T2.

All her findings were suggestive of Pott’s spine. The patient was advised surgery but she denied it. So, a halo vest was applied, chemotherapy was started, and the patient was made aware of the red flags. Two weeks later, the patient returned with a complete neurological deficit.

**Figure 3:**
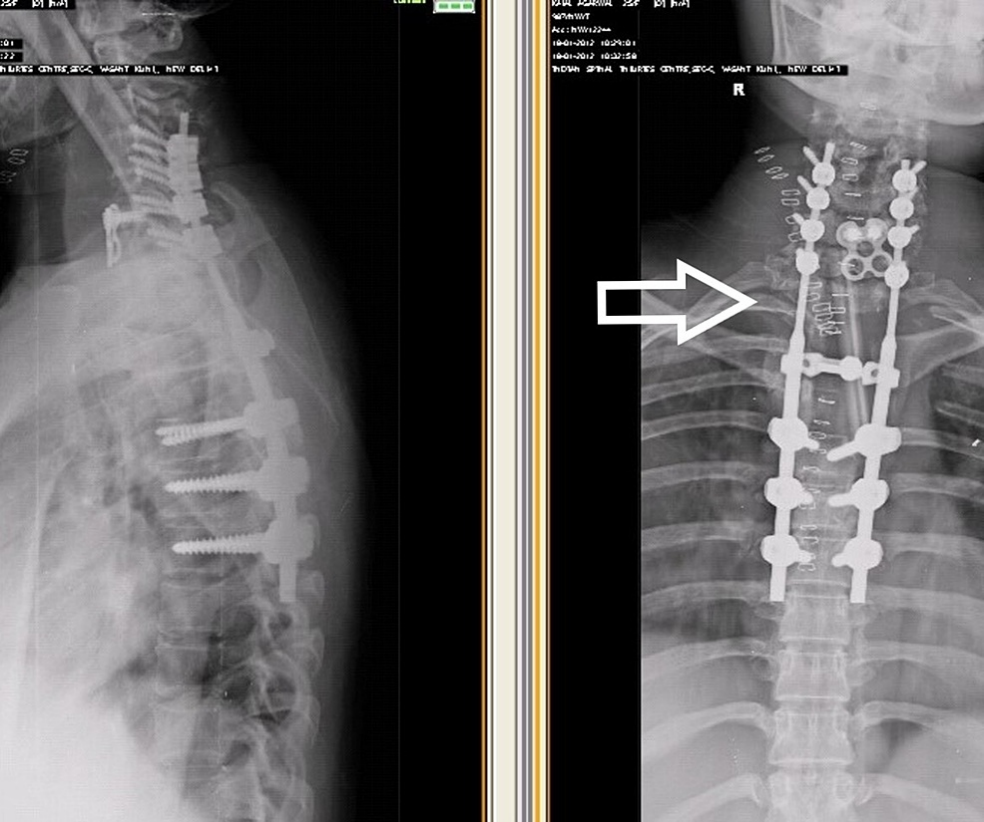
Immediate post-operative X-ray

We did a two-stage procedure in a single sitting (Figure 3). In the first stage, with the anterior cervical approach, T1 corpectomy and plate were applied. For the second stage, the patient was flipped and C7-T4 laminectomy, T2,3,4 corpectomy, and application of fibular autograft between C6 and T5 with posterior stabilization from C4-T7 was done. The procedure went well, and post-operatively, the patient showed full recovery of neurological deficit in six months.

**Figure 4:**
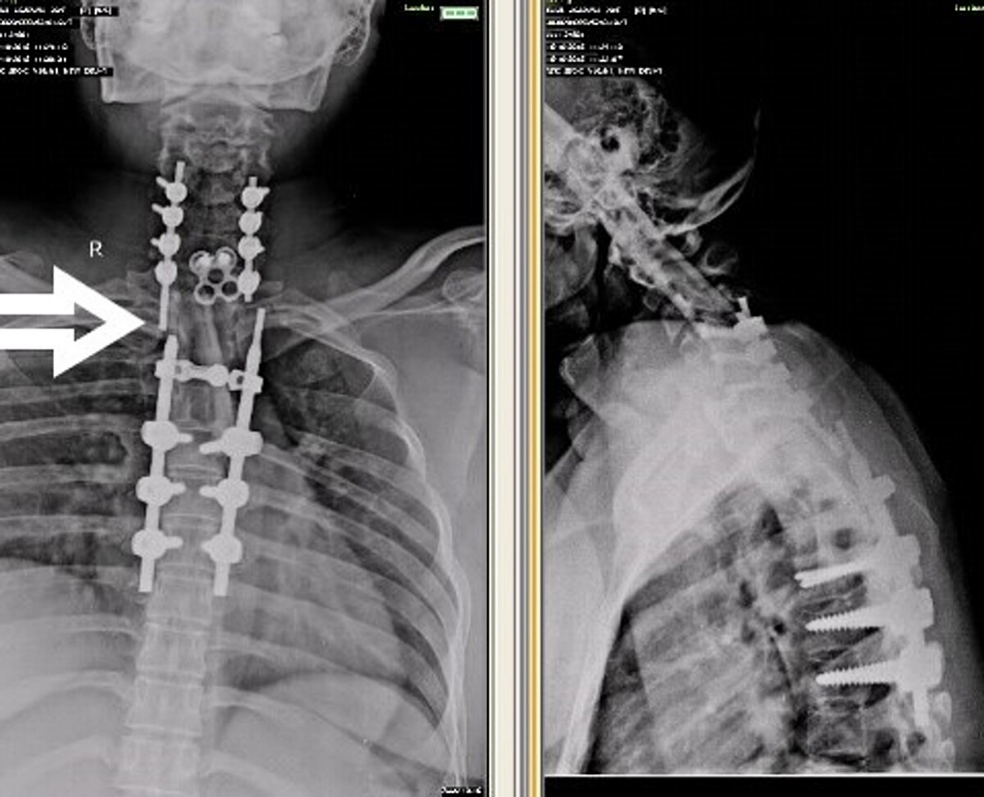
X-ray after four years of surgery showing breakage of both rods

**Figure 5:**
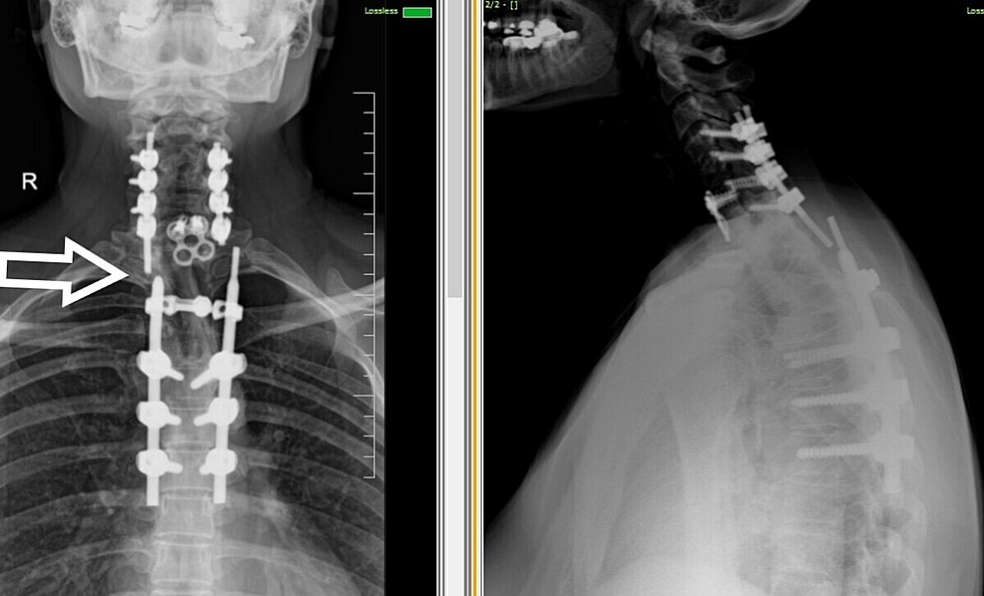
X-ray after seven years with no change in implant status

**Figure 6:**
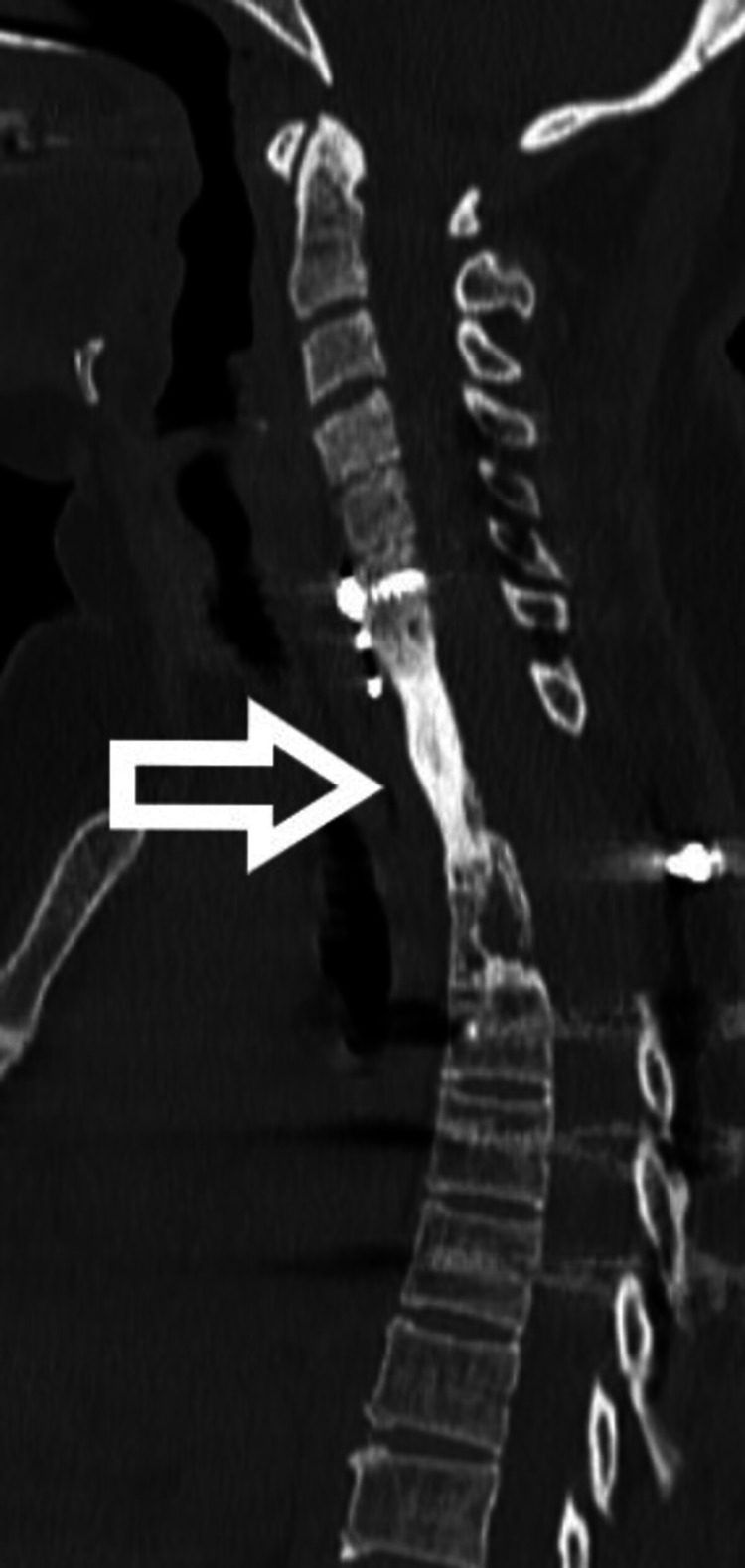
CT scan shows solid incorporation of fibular graft

Chemotherapy was given for 12 months. At 2.5 years follow-up, the patient showed breakage of the left-side rod with no fresh complaints and neurology was normal. At 4 years follow-up, both rods were broken (Figure 4). Since the patient was asymptomatic and there was no neurological deficit, we decided to keep her under observation, and the patient was educated for red flag signs. At 7 years follow-up (Figure 5), the patient is asymptomatic and X-ray shows no further change in implant status. On CT scan (Figure 6), bony fusion can be appreciated at the corpectomy site.

Case 2

A 55-year-old female presented to us with back pain and claudication pain in both lower limbs.

**Figure 7:**
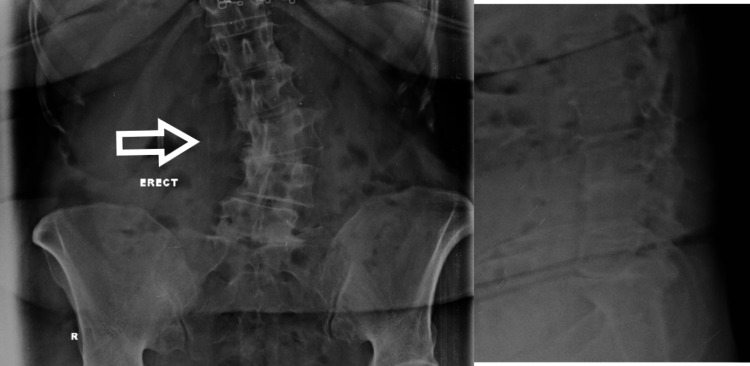
Pre-operative X-ray showing degenerative scoliosis

**Figure 8:**
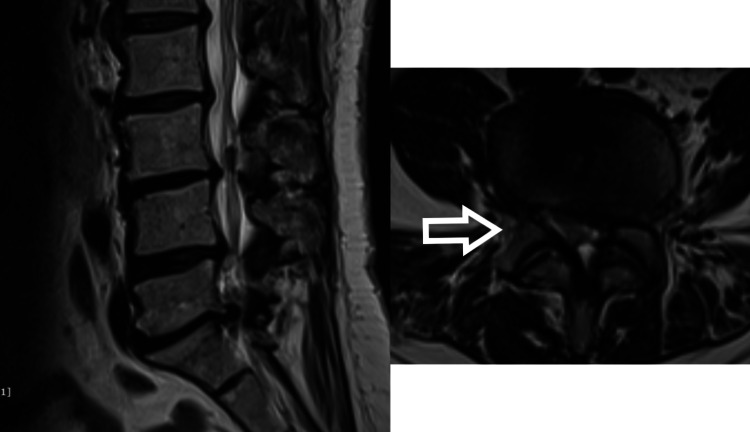
Pre-operative MRI showing Lumbar canal stenosis responsible for claudication symptoms

She was evaluated and found to have degenerative scoliosis with malalignment of global sagittal and coronal balance with lumbar canal stenosis (Figures 7, 8). So, she was managed surgically after the failure of an appropriate conservative trial.

**Figure 9:**
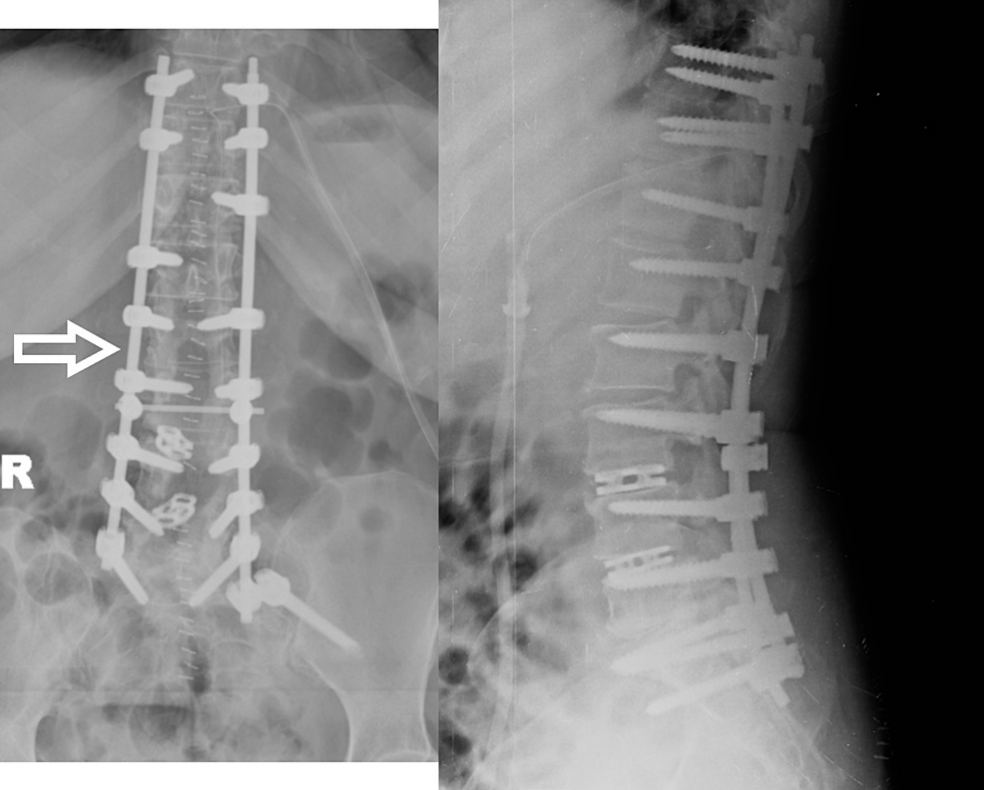
Immediate post-operative X-ray

She was instrumented from T10 to pelvis and interbody fusion was done at multiple levels using the standard posterior approach (Figure 9). She was doing fine post-operatively and was kept on regular follow-up.

**Figure 10:**
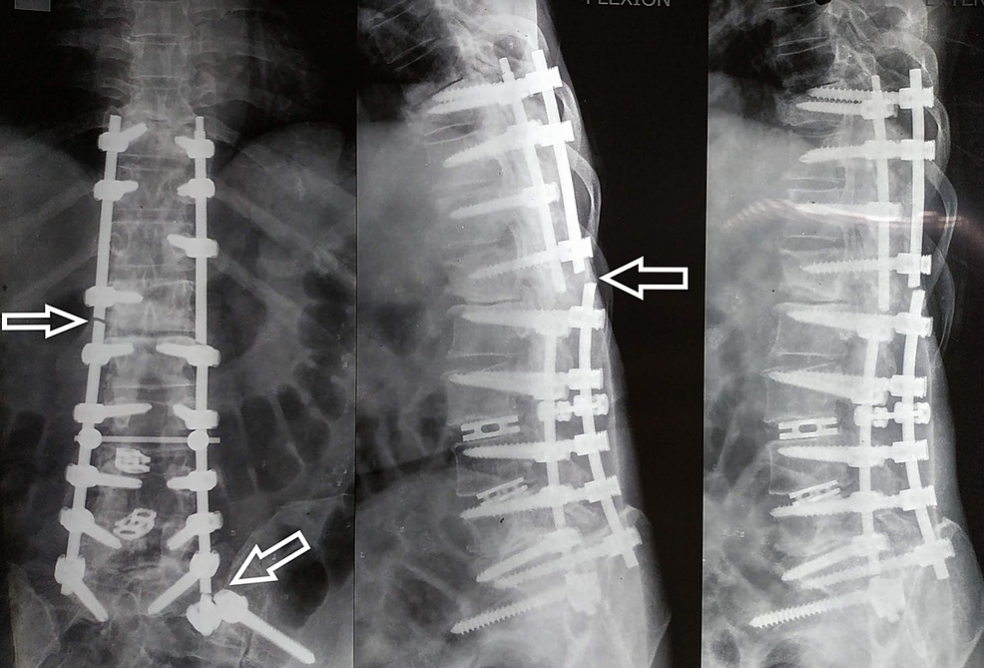
X-ray with anteroposterior and dynamic lateral views after five years of presentation with rod breakage

**Figure 11:**
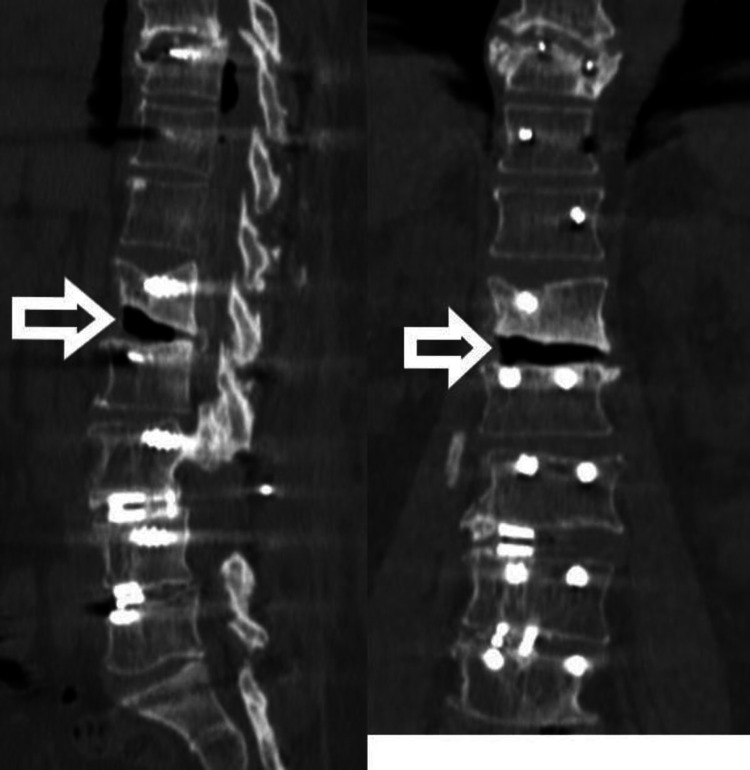
CT scan showing “vacuum sign” at the site of rod breakage

After six years, she came with generalized back pain. There were no symptoms of nerve compression. She was evaluated clinically and radiographically. On X-ray, we could see there was a breakage of both rods at the dorso-lumbar junction (Figure 10). Also, there was a breakage of the left side rod between the S1 screw and the S2-iliac screw. On CT scan, “vacuum phenomenon” can be seen in the disc space corresponding with the site of breakage of the rod (Figure 11). The solid bony union can be appreciated in the rest of the construct. She was managed conservatively with trigger point injections, analgesics, and physiotherapy. After five years of follow-up with conservative management, the patient was completely asymptomatic.

## Discussion

Among all the complications, implant failure has always been the deadliest complication in spine surgery. Implants are put to support the construct till the time there is natural bony fusion. In earlier days, the incidence of implant failure was high as the material used for the implant was not optimal [1]. With the advent of better implants and techniques, the incidence has greatly reduced. The incidence of non-mechanical causes of surgical failure like infection and wound dehiscence are mostly seen within one year of surgery. Mechanical causes like pseudoarthrosis, implant failure, and junctional failure have been reported to occur between two years and five years [5]. The ultimate aim of all the instrumented spine surgeries is fusion. With the development of pseudoarthrosis, there is continued stress on implants which inevitably leads to instrumentation fracture or loosening [3]. According to the definition, implant failure can be said if it ceases to perform the function for which it is inserted. Most of the authors in the literature consider implant breakage the same as implant failure and both the terms are loosely used interchangeably. Implant breakage is one of the most common presentations of pseudoarthrosis, but not all implant breakage should be considered as implant “failure”.

The incidence of pseudoarthrosis in spine surgery varies from 3 to 83% [6], considering the different figures mentioned by different authors. This disparity is related to the diagnosis of pseudoarthrosis, surgical approaches, and instrumentation types. The incidence of pseudoarthrosis is more commonly seen at cervicothoracic, thoracolumbar, and lumbosacral regions due to stress concentration and transition which lead to increased tensile and shear forces on implants and fusion mass [4]. There are various risk factors for the development of pseudoarthrosis, which include patient and procedure-related factors and is beyond the scope of this article. Cost-effective analyses of reoperation for pseudoarthrosis show the high and variable cost of such procedures [7, 8], which is a great burden, especially in developing countries.

There are no single consistent guidelines available for the diagnosis of pseudoarthrosis. The initial investigation in most of the cases is plain radiographs and the most common radiographic finding is rod breakage [4]. But not all patients with implant breakage on radiographs are symptomatic. There are other modalities available like dynamic radiographs and advanced imaging like CT scan. In dynamic radiographs, interspinous movements and changes in Cobb’s angle at adjacent segments can be helpful to diagnose pseudoarthrosis. But this method has shown increased inter- and intra-observer reliability. Also, there are various parameters defined on CT scan including extra-graft bridging bone and intra-graft bridging bone, but the results are inconsistent with other radiographic indicators and not reliable in all cases [9]. In the study published by Peters et al. [10], they found a better technique for the diagnosis of pseudoarthrosis. They concluded that the ^18^F-fluoride positron emission tomography (PET) scan has better accuracy compared to the CT scan in symptomatic patients, but in asymptomatic patients, CT scan is better. Also, ^18^F-fluoride PET is not available everywhere and is expensive. Hence, no investigation is accurate for the diagnosis of pseudoarthrosis, and also no separate and reliable guidelines are available for the cervical, thoracic, and lumbar spine.

After going through all the literature available to date, the only confirmed way to diagnose pseudoarthrosis is a surgical exploration, which is not feasible in every case as most of the patients are asymptomatic. This can also be seen in the study of Hofler et al. [8], which claims reoperation rates in cases of pseudoarthrosis to be 1.2% after cervical fusion and 1.8% after thoracic and lumbar fusion. However, when the authors compared their reoperation rates with other similar studies of Shriver et al. and How et al. [9, 11], they found lower rates of reoperation. On looking into the possible explanations of their lower incidence compared to other studies, the authors clearly mentioned that their study identified the patients having clinically meaningful pseudoarthrosis, whereas other studies involve patients only based on radiographic parameters. Hence not all implant breakage can be called as a failure of surgery or implant “failure”.

To the best of our knowledge, there is only one study [12] available that shows the five-year follow-up of non-operative management of pseudoarthrosis of 19 patients presented with rod breakage after pedicle subtraction osteotomy. In this study, all patients presented with either single or both rod breakage. The authors of this study have compared the self-image subscale and mental health subscale with patients who have undergone revision surgery [13] for pseudoarthrosis. They found no change in the self-image subscale but decreased mental health subscale after revision surgery. The possible explanation for successful non-operative management is maintained sagittal vertical axis during follow-up after breakage of the implant.

In both of our patients, we had similar findings of rod breakage, indicating possible pseudoarthrosis. While the first patient with cervicothoracic Pott’s spine was asymptomatic even after breakage of both the rods, the second patient with degenerative scoliosis had diffuse back pain. We had no difficulty in deciding for non-operative management with frequent follow-ups for the first patient. But after all the imaging modalities and considering the symptoms, there was confusion regarding the possibility of symptomatic pseudoarthrosis in the second patient. We took advantage of the absence of any neurologic symptoms and decided to manage her conservatively. Both the patients did well, one at seven years and the other at five years follow-up.

Since the patients presented with implant breakage and investigations were not reliable in diagnosing pseudoarthrosis accurately, the term “implant failure” cannot be convincingly used in such cases. More, well-organized, randomized studies are required in this field to establish the guidelines for the role of conservative or non-operative management in patients presenting with implant breakage. This may reduce the financial and mental health burden of reoperation in cases of implant breakage.

There are several limitations to this study. First, this is a retrospective study. Second, only two patients are taken into consideration. Third, there is no comparison group. In addition, it is unclear whether higher follow-up would impact our findings. Also, both the patients are of separate regions of the spine.

## Conclusions

Our study advocates that not all patients with implant breakage require surgery. If the patient has no neurological symptoms, then non-surgical treatment can be advised.

In resource-poor countries like India, conservative treatment should be given priority. More studies with a larger sample size and longer follow-up are required to formulate proper guidelines of conservative and surgical management after implant breakage.
